# A longitudinal study of mental health before and during COVID-19 lockdown in the French population

**DOI:** 10.1186/s12992-021-00682-8

**Published:** 2021-03-22

**Authors:** Leila Ramiz, Benjamin Contrand, Madelyn Yiseth Rojas Castro, Marion Dupuy, Li Lu, Catherine Sztal-Kutas, Emmanuel Lagarde

**Affiliations:** 1 Institut de Santé Publique, d’Epidémiologie et de Développement (ISPED), Université de Bordeaux, Bordeaux, France; 2 Team IETO, Bordeaux Population Health Research Center, UMR U1219, INSERM, Université de Bordeaux, Bordeaux, France; 3 Calyxis, Centre of risk expertise, Niort, France

**Keywords:** Anxiety symptoms, COVID-19, Depression symptoms, Lockdown, Mental health

## Abstract

**Background:**

The impact of general population lockdown implemented in the face of the COVID-19 epidemic needs to be evaluated. We describe here a longitudinal study on the mental health of adults in France.

**Methods:**

We did a secondary analysis of a web-based cohort, initially set up to study home and leisure injuries, in order to measure the consequences of the national lockdown implemented in France from 17 March 2020 to 11 May 2020, and to assess potential vulnerability and resilience factors. Eligible participants were invited to answer an online questionnaire designed to assess their living conditions and health during lockdown. Comparisons were done with answers provided 4.8 years earlier on average.

**Results:**

On 15th April 2020, we sent email invitations to 9598 participants recruited between November 2014 and December 2019 and 1237 volunteers took part in the study by completing the online questionnaire. The proportion of those with anxiety symptoms markedly increased from 17.3 to 20.1%. The average self-rated level of mental health decreased from 7.77 to 7.58. Women, the elderly and the youngest appeared to be more vulnerable. A small living space (less than 30 m^2^) was associated with an increase in depression symptoms (PHQ-9 score), and poorer self-rated physical health at recruitment was associated with an increase in anxiety symptoms (GAD-7 score). On the contrary, the average self-rated level of physical health markedly increased from 7.44 to 7.94 between recruitment and lockdown, and the proportion of those who reported a level of 9 or 10 jumped from 25.7% at recruitment to 43.1% during lockdown.

**Conclusions:**

Mental health deteriorated during lockdown in France during the 2020 COVID-19 crisis. Overall, self-rated physical health improved but those who experienced a worse physical health were more likely to report anxiety symptoms.

## Introduction

Among the measures chosen to avoid a massive spread of the pandemic of SARS-CoV-2 is the implementation of lockdown. Most countries have used population lockdown to limit the spread of the SARS-Cov-2. While this may have emerged as a necessary evil, the scientific evidence already available shows that it is not without consequences from a mental health perspective.

The psychological impact of quarantine has been studied during past epidemic outbreaks [1], and includes depression, stress, irritability, insomnia, anxiety, poor concentration, indecisiveness and post-traumatic stress disorders. These observations correspond, however, to the situation of persons placed in solitary confinement on an individual basis. The situation experienced during the COVID-19 crisis is different, since several billion individuals had to stay at home for several weeks. Isolation has been compounded by concerns about the threats posed by the epidemic itself and the need to live with relatives and friends for long periods of time and often in a limited space.

In France, the entire population was placed under mass quarantine for 8 weeks, from 17 March 2020 to 11 May 2020. People were only allowed to leave their homes for proven necessities, such as health reasons and basic necessities and to work for those who could not work at home.

A large number of studies are now being carried out on the mental health consequences of the current COVID-19 epidemic. Many of them have compared the level of various mental health indicators measured in cross-sectional surveys conducted in the general population [2–7]*,* pregnant women [8], children with physical disabilities [9], lesbian, gay, bisexual, and transgender adults [10], college students [11], and university students [12]. All of them show a clear degradation of mental health during lockdown, linked to the anxiety caused by the epidemic but also to poor living conditions. Longitudinal cohort studies are much less frequent: a study of 217 undergraduate students in the US described their behaviour and health during lockdown. A much larger secondary analysis of the UK household longitudinal study measured the deterioration of the mean General Health Questionnaire score [13]. Another longitudinal study of 1442 students in health professions at Sichuan University, China was more focused on acute stress reactions than on general mental health [14]. Finally, Shanahan and colleagues conducted a longitudinal study which showed the impact of the pandemic on stress among 786 participants [15].

In order to assess whether the COVID-19 crisis and the national lockdown implemented in France from 17 March 2020 to 11 May 2020 have had a negative impact on depressive and anxiety symptoms, we proposed to volunteers previously enrolled as part of the MAVIE cohort to report their mental health status and symptoms related to anxiety and depression and we assessed potential vulnerability and resilience factors.

## Methods

### Study design and recruitment

The MAVIE cohort is a web-based prospective cohort study, with a longitudinal follow-up of home, leisure, sports, and school injuries.

All households in France and in French overseas territories are eligible to participate. The recruitment process began in November 2014 and is still ongoing.

Cohort management is entirely online, including invitations, registration, and data collection. The largest share of participants was recruited through an email invitation sent to their insurees by three mutual insurance companies (MAAF, MACIF, and MAIF). A smaller proportion of the participants were informed of the MAVIE cohort and invited to participate through press releases, social media, posters, and flyers. No incentives were offered for participation.

Potential participants are asked to choose a household reference member, who receives all correspondence and reminder messages and is in charge of reporting home and leisure injuries (HLIs) that may happen to any consenting household members. Each member was free to participate or not and had to provide individual consent.

The criteria for inclusion in the cohort were: 1) residing in France, 2) being able to answer the questionnaires in French, 3) having access to and being able to use the internet (at least the reference member). In an attempt to address the foreseeable underrepresentation of older people who may have difficulties using computers, another participation status was created for caregivers whose only role is to represent older persons. It was also possible to participate to represent a child.

**Fig. 1:**
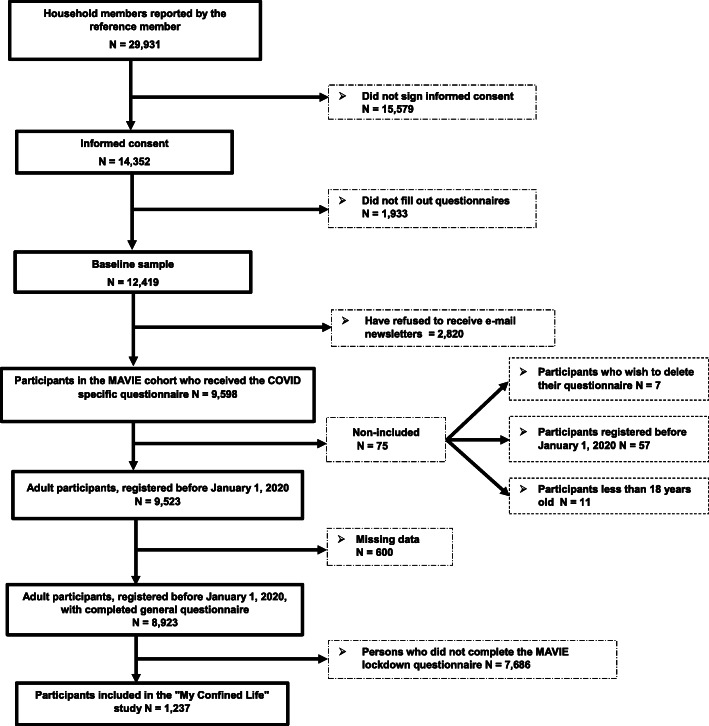
Flowchart of the inclusion procedure

Between November 2014 and December 2019, 14,352 people signed a consent form to participate in the study (Fig. 1) and then were asked to complete several web-based questionnaires designed to provide information on individual variables concerning sociodemographic characteristics, health, domestic, sport and leisure activities, and lifestyles. Each reference member was also asked to provide information on the characteristics of his or her home. The volunteers have been prospectively followed for an average of 4.8 years.

On April 15, 2020, a specific questionnaire was designed and proposed to all active volunteers in the cohort to describe their living conditions and health during lockdown (the “lockdown questionnaire”). Minors, those participating in the study through a third party, and participants registered after 1st January 2020 were not invited to participate in the study. Participants included in the study were those who have completed the questionnaire between April 15, 2020 and May 4, 2020.

### Data selection

#### Variables selected from the MAVIE cohort database

We selected the following variables for the present study, all of them reported at inclusion in the MAVIE cohort: gender, age, marital status, highest education degree obtained, type of work, employment status, monthly household income, typology of the residential area, size of the municipality, type of housing (house, apartment). We also selected data on self-reports related to the participants’ activities (on-screen, gardening, crafts, etc.) and to health problems and disabilities.

Self-perceived mental health and self-perceived physical health, were both self-rated using a visual analogue scale (1 = *Poor health*, 10 = *Excellent health*). Depression symptoms were collected using the *Patient Health Questionnaire-9* (PHQ-9), which is a validated measure in the general population [16, 17]. Participants indicated how often they have been bothered by each symptom over the last 2 weeks using a four-point Likert scale ranging from 0 (*Not at all*) to 3 (*Nearly every day*), summing up to an overall score that ranges from 0 to 27. Scores of 5,10 and 15 are regarded as the cut-off points for mild, moderate and severe depression symptoms. We considered those having a score higher than 4 as with possible depression. Anxiety symptoms were measured using the *Generalized Anxiety Disorder 7-item Scale* (GAD-7), its use in the general population is also validated [18, 19]. Participants indicate how often they have been bothered by each symptom over the last 2 weeks on a four-point Likert scale (0 = *Not at all*, to 3 = *Nearly every day*). The overall score then ranges from 0 to 21. Scores of 5, 10, and 15 are regarded as the cut-off points for mild, moderate and severe anxiety symptoms respectively. We considered those having a score higher than 4 as with possible anxiety.

### Lockdown questionnaire

#### Mental health, physical health, anxiety and depression symptoms

The four sets of questions on self-rated mental health, self-rated physical health, depression symptoms (PHQ-9) and anxiety symptoms (GAD-7) were repeated in the lockdown questionnaire. The differences between the levels reported during lockdown and the levels reported at recruitment were our main outcomes.

#### Living conditions and sociodemographics

Questions were devoted to the collection of data on the current living environment during lockdown, including possible change of residence, the size of the dwelling, the number of people sharing it, if this included children, and the availability of outside space. A series of questions provided information on working conditions and the possible use of teleworking.

#### Perceptions and experience of the epidemic and lockdown

Participants were asked whether they or a relative had been affected by COVID-19, either as a known or suspected infection. They were also asked to use a visual analogue scale (1 = *Least worrying*, 10 = *Most worrying*) to estimate the impact of lockdown on their personal life, family and loved ones, their financial situation and on the country in general.

#### Activities during lockdown

Activities during lockdown could be reported, including the time spent on traditional media (television, radio, and printed press), the internet and social media.

### Statistical analyses

Data analyses were conducted in three steps.

(i) We first compared the main sociodemographics between respondents and non-respondents to the lockdown questionnaire.
(ii) Secondly, we assessed changes in self-rated mental health level, anxiety symptoms as measured with GAD-7 score, and depression symptoms as measured with PHQ-9 score between recruitment and lockdown. We coded three corresponding Boolean variables to indicate whether there was an increase in the level of self-rated mental health or a decrease in the GAD-7 score and in the PHQ-9 score. We fitted a first set of logistic models to assess the association between baseline participants’ characteristics and the three latter binary outcomes.
(iii) In order to assess how changes in mental health were related to the COVID-19 crisis and lockdown, we tested potential correlations between the same set of three Boolean variables and self-reported concerns or difficulties associated with the virus threat or lockdown listed in the lockdown questionnaire. We fitted three separate models (mental health, anxiety symptoms, depression symptoms) adjusted for the variables found to be significant in step (ii).

For both step (ii) and (iii), a full multivariate logistic regression models was fitted and variables that proved not significant (*p* > 0.05) were excluded one by one. Data were analysed using SAS 9.4 (SAS Institute, Cary, NC, USA).

### Ethics, confidentiality and individual data protection

The French Data Protection Authority approved the protocol of the MAVIE cohort, declared to the CNIL under the file number 912292. Identifying data (name, surname, and email address) were stored on servers located in a different location from those hosting the main database. Electronic informed consent was collected from all adult participants. The participation of children was done under the responsibility and with the consent of a legal guardian.

## Results

### Participants

On 15th April 2020 we sent 9598 invitation emails to the eligible participants (Fig. 1), and a total of 1237 people took part in the study by completing the online lockdown questionnaire.

Compared to non-respondents, participants who completed the lockdown questionnaire were older and more likely to be retired (Table 1). Respondents were also in better health, either mentally or physically, and reported less sever levels of anxiety and depression symptoms than non-respondents at inclusion. Respondents smoked less but reported greater alcohol consumption.

**Table 1:** Comparison of respondents and non-respondents

	Respondents		Non-respondents		
	***n*** **= 1237**		***n*** **= 7686**		
	**n**	**%**	**n**	**%**	***p*-value**
**Gender (*n*** **= 8923)**					0.7
Male	601	48.6	3688	48.0	
Female	636	51.4	3998	52.0	
**Age (n = 8923)**	Mean 61.8	SD 12.7	Mean 58.9	SD 14.4	**< 10**^**−4**^
**Marital status (*n*** **= 7044)**					0.4
As a couple (marriage. Civil union. Cohabitation with a spouse/partner)	852	71.4	4161	71.1	
Divorced or separated	143	12.0	779	13.3	
Widowed	50	4.2	199	3.4	
Single	145	12.1	688	11.8	
Other	3	0.3	24	0.4	
**Highest education obtained (*n*** **= 7053)**					**< 10**^**−4**^
Less than General Baccalaureate	288	24.1	1781	30.4	
General Baccalaureate or Diploma level BAC + 2	329	27.6	1646	28.1	
Diploma level BAC + 3 or higher	576	48.3	2433	41.5	
**Monthly household income (*n*** **= 6610)**					**< 10**^**− 4**^
Less than €2500	280	27.3	1889	33.8	
From 2500 to less than 4167 €.	376	36.6	2182	39.1	
€4167 and more	370	36.1	1513	27.1	
**Place of residence (*n*** **= 7135)**					**< 10**^**−3**^
Rural area (municipality with less than 5000 inhabitants)	392	35.3	2470	41.0	
Municipality with 5000 to 30,000 inhabitants	334	30.1	1759	29.2	
Municipality of more than 30,000 inhabitants	385	34.6	1795	29.8	
**Housing type (*n*** **= 7288)**					0.78
Detached house	781	69.7	4271	69.3	
Apartment	340	30.3	1896	30.7	
**Number of rooms in the dwelling (*n*** **= 7274)**					**< 0.05**
One to four rooms	239	21.4	1494	24.3	
Five or six rooms	437	39.0	2439	39.6	
Seven or more rooms	443	39.6	2222	36.1	
**Size of living space (in m**^**2**^**) (*n*** **= 7202)**	Mean113.1	SD 49.3	Mean 112.4	SD 54.4	0.64
**Personal outdoor space (garden. Land. courtyard. ...) (*n*** **= 6980)**					0.11
No	272	25.1	1620	27.5	
Yes	812	74.9	4276	72.5	
**Tobacco consumption at recruitment (*n*** **= 6071)**					**< 10**^**−4**^
Smoker	108	9.4	782	15.9	
Ex-smoker	285	24.9	1064	21.6	
Non-smoker	752	65.7	3080	62.5	
**Frequency of alcohol consumption at recruitment (*n*** **= 6088)**					**< 10**^**−3**^
Never	97	8.5	532	10.8	
Once a month or less often	179	15.6	904	18.3	
2 to 4 times a month	346	30.2	1458	29.5	
2 to 3 times a week	216	18.8	977	19.8	
4 times a week or more often	309	26.9	1070	21.6	
**Frequency of holidays (*n*** **= 6428)**					**< 10**^**−2**^
Two or more times a year	567	49.1	2307	43.7	
Once or twice a year	391	33.8	1806	34.3	
Only once in two years	91	7.9	509	9.6	
Only a few weekend outings	55	4.8	362	6.9	
Never	51	4.4	289	5.5	
**Regular activities**					
With a screen (*n* = 6475)	1108	95.7	4937	92.8	**< 10**^**−3**^
Reading (*n* = 6470)	1001	86.7	4262	80.2	**< 10**^**−4**^
Indoor activities (*n* = 6446)	238	20.8	1092	20.6	0.89
Outdoor activities (*n* = 6432)	67	5.9	451	8.5	**< 10**^**−2**^
Gardening (*n* = 6455)	649	56.5	2907	54.8	0.31
DIY (*n* = 6450)	570	49.7	2562	48.3	0.41
Going out (*n* = 6460)	752	65.3	3189	60.1	**< 10**^**−3**^
Manual activities (*n* = 6440)	199	17.4	846	16.0	0.25
Sporting activities (n = 6470)	932	80.8	3970	74.7	**< 10**^**−4**^
**Self-perceived mental health status (*n*** **= 6271)**					**< 10**^**−2**^
1 to 7	420	36.6	2112	41.2	
8	301	26.2	1306	25.5	
9 to 10	428	37.2	1704	33.3	
Mean (SD)	7.75	(1.7)	7.54	(1.8)	**< 10**^**−3**^
**Self-perceived physical health status (*n*** **= 6269)**					**< 0.05**
1 to 7	500	43.6	2479	48.4	
(8	354	30.8	1437	28.1	
9 to 10	294	25.6	1205	23.5	
Mean (SD)	7.44	(1.7)	7.24	(1.7)	**< 10**^**−3**^
**Anxiety symptoms GAD-7 score (*n*** **= 5467)**					**10**^**−3**^
≤ 4	860	82.7	3442	77.8	
> 4	180	17.3	985	22.2	
**Depression symptoms PHQ-9 score (*n*** **= 5356)**					**< 10**^**−2**^
≤ 4	752	73.0	2934	67.8	
> 4	278	27.0	1392	32.2	

The participants were between 23 and 93 years of age, with a mean age of 62 years (SD =12.7). Most of the participants were retired (58%) or pre-retired (20%). The average scores of self-rated mental and physical health status were 7.56 (SD = 1.9) and 7.94 (SD = 1.76).

Respondents reported a low average impact of the pandemic on their financial situation, with a mean of 3.71 (SD = 2.7) on the 1 to 10 scale. On the other hand, the estimated negative impact on the country as a whole was judged considerably higher with a mean of 8.20 (SD = 1.9). The mean ratings for the impact on the participants’ personal life, and on family and relatives, were 5.61 (SD = 2.5) and 6.38 (SD = 2.3), respectively. Most participants did not change residence (95%), and many had an outdoor space for personal use during lockdown (82%). Only 23% of households included at least one child.

A total of 261 people in the participants’ circle of family and friends were reportedly diagnosed positive for COVID-19 infection, and 267 had suspected infection. Only 39% of the participants judged lockdown as moderately to extremely disruptive and only 10% did not find it disruptive at all. Participants reported to spend a little more than 2 h in average a day to obtain information concerning epidemic information (TV, newpapers, radio,website) (Table 2).

**Table 2:** Data collected by the COVID-19 questionnaire (1237 respondents)

	n	Proportion (%)	Mean	SD
Number of persons at home during lockdown (*n* = 1163)			2.2	1.1
Size of living space during lockdown (in m^2^) (*n* = 1181)			113.9	48.3
**Media (in minutes per day) (n = 1237)**				
Time spent on traditional media for epidemic information			91	92
Time spent on websites for epidemic information			36	51
Time spent on social media			60	84
**Pandemic’s impact (Between 0 and 10)**				
On personal life (*n* = 1157)			5.6	2.5
On family and loved ones (*n* = 1151)			6.4	2.3
On personal financial situation (*n* = 1155)			3.7	2.7
On the country in general (*n* = 1156)			8.2	1.9
**Change of residence (*n*** **= 1189)**				
No. Without taking in relatives	1073	90.2		
No. Taking in relatives	59	5.0		
Yes. Going to a second home	24	2.0		
Yes. Going to relatives	23	1.9		
Other	10	0.9		
**Layout of a personal outdoor space during lockdown (*n*** **= 1190)**				
No	219	18.4		
Yes	971	81.6		
**Life during lockdown (*n*** **= 1191)**				
Alone without children	273	22.9		
Alone with child (ren)	43	3.6		
In a couple without children	593	49.8		
In a couple with child (ren)	235	19.7		
Roommates. With friends	1	0.1		
With parents. Family members	34	2.9		
Other	12	1.0		
**Work situation during lockdown (n = 1189)**				
Unemployment	32	2.7		
At home	17	1.4		
Activity currently suspended	63	5.3		
Currently reduced activity	43	3.6		
Activity maintained by teleworking	233	19.6		
Unchanged activity (goes to work)	70	5.9		
Work on a voluntary basis	4	0.3		
Student with continuing distance learning	3	0.2		
Student whose training is suspended	0	0		
Retired or pre-retired	688	57.9		
Disability or long-term illness	9	0.8		
Other	27	2.3		
**Clinical diagnosis or positive COVID-19 test (Multiple-choice question)**				
No	917			
The participant	8			
Family members	106			
Close friends	51			
Acquaintance	143			
**COVID-19 suspicion (Multiple-choice question)**				
No	914			
The participant	34			
Family members	111			
Close friends	56			
Acquaintance	123			
**Acceptance of COVID-19 vaccine (*n*** **= 1183)**				
Yes	969	81.9		
No	214	18.1		
**Evolution of perceived mental health during lockdown (*n*** **= 1085)**				
Decrease	422	38.9		
Stable	296	27.3		
Increase	367	33.8		
**Evolution of self-perceived physical health during lockdown (*n*** **= 1083)**				
Decrease	257	23.7		
Stable	294	27.2		
Increase	532	49.1		
**Evolution of depression symptoms (PHQ-9 score) during lockdown (*n*** **= 954)**				
Decrease	140	14.7		
Stable	660	69.2		
Increase	154	16.1		
**Evolution of anxiety symptoms (GAD-7 score) during lockdown (*n*** **= 958)**				
Decrease	98	10.2		
Stable	733	76.5		
Increase	127	13.3		
**Lockdown experience (*n*** **= 1161)**				
Not at all disturbing	122	10.5		
A little disturbing	584	50.3		
Moderately disruptive	322	27.7		
Highly disruptive	120	10.4		
Extremely disruptive	13	1.1		
**Request for professional help (*n*** **= 1164)**				
Yes	37	3.2		
No	1127	96.8		
**Smoking during confinement (*n*** **= 1166)**				
Smoker	74	6.4		
Ex-smoker	265	22.7		
Non-smoker	827	70.9		
**Frequency of alcohol consumption during lockdown (*n*** **= 1167)**				
Never	152	13.0		
Once a month or less often	218	18.7		
2 to 4 times a month	279	23.9		
2 to 3 times a week	229	19.6		
4 times a week or more often	289	24.8		
**Time of exposure to epidemic information (*n*** **= 1160)**				
Too much time	214	18.4		
Too much time. Suffering	68	5.9		
Current situation is suitable	820	70.7		
Feelings of not having enough information	58	5.0		

### Perceived health

**Fig. 2:**
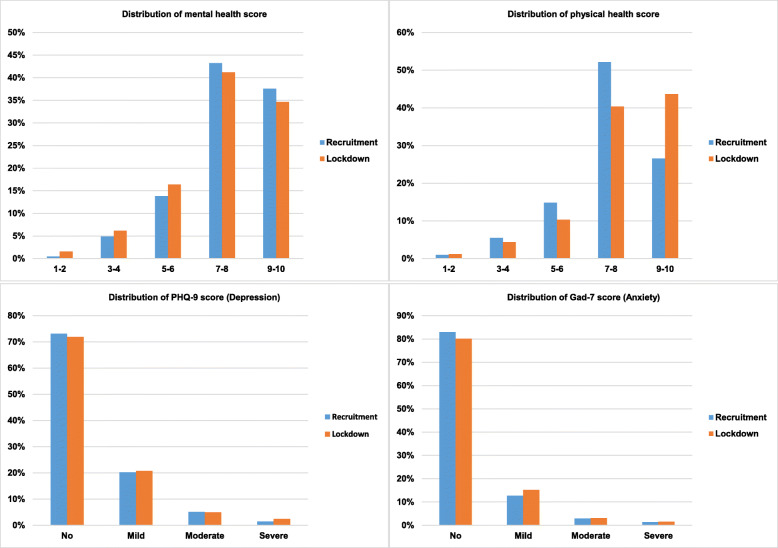
Distribution of mental health, physical health, PHQ-9 and GAD-7 scores

The self-rated levels of health between recruitment and lockdown increased on average from 7.44 to 7.94 (*p* < 10^− 4^) for physical health and decreased from 7.77 to 7.58 (*p* = 10^− 2^) for mental health. Figure 2 shows a clear downward shift in self-rated mental health and the proportion of those who reported a lower and a higher average level of mental health at lockdown than at recruitment was 38.9 and 33.8%, respectively. Conversely, the proportion of those who reported a level of 9 or 10 for physical health jumped from 25.7% at recruitment to 43.1% during lockdown and the proportion of those who reported a lower and a higher average level of physical health at lockdown than at recruitment was 23.7 and 49.1%, respectively.

### Anxiety and depression symptoms

The proportion of those considered with possible depression (PHQ-9 score > 4) remained unchanged from 27.0% at recruitment to 27.6% at lockdown. The proportion of those considered with possible anxiety (GAD-7 score > 4) markedly increased from 17.3 to 20.1%, with 13.3% of the respondents scoring higher at lockdown (see Fig. 2).

The increase in depressive symptoms appeared to be significantly greater for those who responded to the questionnaire at the end of lockdown (20.1% for April 21- to May 6) than at its beginning (14.6% for April 14 to April 20).

### Characteristics measured at recruitment and associated with impaired mental health

The assessment of background and living condition variables associated with a decreased self-rated level of mental health showed that those aged 23–49 and those aged 70 and over were more vulnerable (Table 3). People with no outdoor space and who reported more than 3 h per week of outdoor time before lockdown were significantly more likely to report a decreased mental health status at lockdown (ORs were 1.38 and 1.47, respectively). Those who used to spend more than 1 hour by day on screen had a lower risk of decrease mental health level at lockdown (OR was 0.65).

**Table 3:** Background and pre-lockdown living conditions as predictors of a decreased self-rated mental health, and of an increase in depression symptoms PHQ-9 score and in anxiety symptoms GAD-7 score. Result from logistic regression analysis

	# participants (% with decreased mental health status score)	Adjusted OR^a^ [95% CI]	# participants (% with increase in depression symptoms PHQ-9 score)	Adjusted OR^a^ [95% CI]	# participants (% with increase in anxiety symptoms GAD-7 score)	Adjusted OR^a^ [95% CI]
**Total**	1085 (38.9)		954 (16.1)		958 (13.3)	
**Gender**						
Male	539 (35.4)	Ref	480 (11.2)	Ref	478 (10.0)	Ref
Female	546 (42.3)	1.25 [0.95–1.63]	474 (21.1)	1.78 [1.20–2.65]	480 (16.5)	1.78 [1.18–2.68]
**Age**						
[23–49]	211 (46.0)	1.74 [1.19–2.54]	186 (24.7)	2.04 [1.17–3.57]	185 (16.2)	1.42 [0.80–2.52]
[50–59]	196 (35.7)	1.10 [0.75–1.63]	172 (14.5)	1.23 [0.67–2.23]	169 (13.0)	1.19 [0.65–2.19]
[60–69]	329 (33.4)	Ref	291 (10.3)	Ref	294 (9.5)	Ref
70 and over	349 (41.5)	1.52 [1.10–2.11]	305 (17.4)	1.83 [1.10–3.02]	310 (15.2)	1.71 [1.03–2.85]
**Lives alone**						
Yes	242 (39.7)		208 (22.1)	1.78 [1.16–2.72]	217 (16.1)	
No	816 (38.2)		723 (14.1)	Ref	717 (12.6)	
**Personal outdoor space**						
Yes	879 (37.0)	Ref	779 (15.1)		775 (12.3)	
No	204 (47.5)	1.38 [1.00–1.89]	173 (20.8)		182 (17.6)	
**Size of living space**						
30m^2^ per person	150 (44.0)		138 (25.4)	1.98 [1.19–3.30]	137 (14.6)	
More than 30m^2^ per person	903 (37.5)		788 (14.1)	Ref	793 (13.0)	
**Self-perceived physical health at recruitment**						
1 to 7	469 (36.5)		410 (18.0)		404 (17.8)	3.31 [1.84–5.95]
8	335 (39.4)		307 (16.9)		298 (13.1)	2.47 [1.32–4.62]
9 to 10	278 (42.8)		231 (11.3)		248 (6.0)	Ref
**Self-perceived mental health at recruitment**						
1 to 7			348 (19.3)		336 (14.9)	
8			248 (14.5)		251 (15.5)	
9 to 10			352 (13.9)		365 (10.1)	
**Weekly time spent outdoors**						
≤ 3 h	832 (37.3)	Ref	726 (15.4)		727 (12.9)	
+ 3 h	238 (46.2)	1.47 [1.08–1.99]	216 (19.0)		220 (14.1)	
**Daily screen exposure time**						
≤ 1 h	275 (46.9)	Ref	234 (17.5)		237 (16.5)	
+ 1 h	756 (37.2)	0.65 [0.49–0.87]	679 (16.1)		680 (12.4)	
**Current smoker**						
Yes	100 (40.0)		87 (24.1)		90 (20.0)	
No	969 (39.0)		860 (15.3)		860 (12.7)	
**Housing**						
Apartment	298 (43.3)		256 (18.0)		267 (14.6)	
House	715 (36.9)		637 (15.9)		627 (12.3)	
**Depressive** symptoms **(PHQ-9 > 4)**						
Yes					231 (19.9)	
No					643 (11.4)	
**Anxiety** symptoms **(GAD-7 > 4)**						
Yes			150 (20.0)			
No			733 (15.3)			
**Monthly household income**						
Less than €1.250 per individual	267 (36.3)		245 (21.6)		229 (14.4)	
More than €1.250 per individual	637 (39.4)		555 (14.4)		569 (12.5)	
**Holidays**						
Maximum once in two years	129 (47.3)		117 (23.9)		107 (20.6)	
More	942 (38.0)		826 (15.0)		841 (12.1)	

^a^ Variable selection followed a manual backward selection process

As regard to symptoms of depression and anxiety, female respondents and those aged 23–49 and more than 70 were more likely to score higher at lockdown. Living alone and less than 30 square metres of living space were associated with a higher risk of an increased depression PHQ-9 score (ORs were 1.78 and 1.98). A low self-rated physical health at recruitment was strongly associated with a higher risk of increased anxiety GAD-7 score.

### Lockdown-related and epidemic-related factors associated with impaired mental health

In order to evaluate how strongly the observed trends in mental health were related to the COVID-19 crisis and/or lockdown, we assessed the associations between concerns reported in the lockdown questionnaire and the three mental health indicators considered in this study (Table 4). Strong correlation measures were consistently found, with high risk of anxiety symptoms (and to a lesser extent depression symptoms and self-rated mental health) for those who reported that COVID-19 had an impact on personal life or on family and relatives. Concerns related to the impact on the country as a whole showed lower association for anxiety symptoms and no association for mental health and depression symptoms. Those who were diagnosed with COVID-19 (OR = 2.18) and who reported spending more than 2 h per day on social media (OR = 2.14) were more likely to have increased anxiety symptoms during lockdown.

**Table 4:** Association between COVID-19-related variable reported during lockdown and mental health^a^. Results from logistic regression analysis

	# participants (% with a decreased mental health status score)	Adjusted OR [95% CI]	# participants (% with increase in depression symptoms PHQ-9 score)	Adjusted OR [95% CI]	# participants (% with increase in anxiety symptoms GAD-7 score)	Adjusted OR [95% CI]
**Total**	1085 (38.9)		954 (16.1)		958 (13.3)	
**COVID-19 diagnosis (participant or relatives)**						
Yes	240 (46.7)	1.38 [1.02–1.87]	211 (18.0)	1.12 [0.73–1.72]	210 (21.0)	2.18 [1.43–3.30]
No	845 (36.7)	Ref	743 (15.6)	Ref	748 (11.1)	Ref
**COVID-19 suspicion (participant or relatives)**						
Yes	249 (47.0)	1.38 [1.02–1.88]	215 (21.4)	1.48 [0.98–2.23]	229 (18.3)	1.52 [0.99–2.32]
No	836 (36.5)	Ref	739 (14.6)	Ref	729 (11.7)	Ref
**Daily COVID-19-related news exposure**						
Less than 3 h per day	859 (37.5)	Ref	763 (15.1)	Ref	758 (12.3)	Ref
3 h or more	226 (44.2)	1.41 [1.03–1.93]	191 (20.4)	1.62 [1.04–2.51]	200 (17.0)	1.56 [0.99–2.45]
**Daily social network exposure**						
Less than 1 h	602 (35.7)	Ref	523 (14.5)	Ref	536 (10.1)	Ref
Between 1 h and 2 h	232 (40.9)	1.31 [0.94–1.83]	208 (12.0)	0.73 [0.43–1.24]	207 (15.5)	1.84 [1.12–3.01]
2 h or more	227 (45.8)	1.43 [1.01–2.04]	201 (24.9)	1.53 [0.97–2.42]	200 (20.0)	2.14 [1.31–3.49]
**Impact on personal life**						
Not worrying (1 to 3)	285 (25.3)	Ref	249 (6.8)	Ref	259 (3.9)	Ref
Mildly worrying (4 to 7)	484 (41.1)	1.93 [1.38–2.70]	425 (14.8)	2.43 [1.33–4.43]	426 (9.9)	2.29 [1.11–4.69]
Very worrying (8 to 10)	296 (49.0)	2.69 [1.86–3.89]	262 (27.5)	5.39 [2.95–9.85]	257 (28.4)	8.62 [4.28–17.34]
**Impact on family and relatives**						
Not worrying (1 to 3)	156 (22.4)	Ref	131 (7.6)	Ref	140 (2.9)	Ref
Mildly worrying (4 to 7)	494 (36.8)	1.91 [1.24–2.95]	443 (13.8)	1.75 [0.82–3.71]	440 (8.9)	2.61 [0.90–7.52]
Very worrying (8 to 10)	409 (47.4)	3.01 [1.93–4.69]	357 (22.4)	3.47 [1.65–7.28]	357 (22.7)	8.33 [2.95–23.46]
**Personal financial impact**						
Not worrying (1 to 3)	619 (35.4)	Ref	535 (13.6)	Ref	553 (9.9)	Ref
Mildly worrying (4 to 7)	302 (46.4)	1.57 [1.17–2.12]	270 (18.1)	1.31 [0.86–1.99]	264 (16.3)	1.53 [0.98–2.39]
Very worrying (8 to 10)	143 (39.2)	1.14 [0.77–1.70]	130 (22.3)	1.62 [0.97–2.71]	124 (21.0)	2.34 [1.37–3.99]
**Impact on the country**						
Not worrying (1 to 7)	266 (35.3)	Ref	243 (13.6)	Ref	241 (6.2)	Ref
Very worrying (8 to 10)	799 (40.3)	1.23 [0.91–1.66]	693 (17.2)	1.38 [0.88–2.16]	701 (15.5)	2.51 [1.41–4.45]

^*a*^ Each line presents the results of a separate model. The adjustment variables are those identified in the models presented in Table 2

There was a slight correlation (*R*-square = 0.056) between increased anxiety symptoms and decreased self-rated physical health. Among participants with a lower GAD-7 anxiety score at lockdown, 65.0% reported an improved physical health status as compared to 37.1% among those with a higher GAD-7 anxiety score at lockdown.

## Discussion

This study conducted among volunteers enrolled in a cohort initially designed to study HLIs took advantage of available self-reported mental health indicators collected on average 4.8 years before lockdown and repeated during lockdown. The proportion of those with anxiety symptoms markedly increased from 17.3 to 20.1%. Self-rated level of mental health decreased from 7.77 to 7.58 on average. This increase in mental distress did not affect all groups of participants equally: women, the elderly and the youngest appeared to be more vulnerable. Small living space (less than 30 m^2^) was associated with an increase in depression symptoms, and a low self-rated physical health at recruitment was associated with more severe level of anxiety symptoms. Conversely, the self-rated level of physical health markedly increased from 7.44 to 7.94 on average, and the proportion of those who reported a level of 9 or 10 jumped from 25.7% at recruitment to 43.1% during lockdown.

The availability of mental health indicators before the COVID-19 crisis in the same population provided the opportunity to compare pre and per lockdown levels, which was not possible for most studies published on the same topic. The many potential selection biases of cross-sectional studies preclude the use of inter-study comparison to assess whether health status are indeed poorer. Even if the population of the MAVIE cohort is not free from selection bias, the fact that health status are compared within the same individuals suggests that the observed variations are real. In addition, we compared the socio-demographic characteristics of respondents and non-respondents in search a further selection bias.

The initial aim of our cohort was to study the risks of accidents in everyday life. Drawing on an existing cohort provided us with a wide range of descriptive variables. This allowed us to better apprehend the impact of the context in which lockdown took place, with no variable selection bias as their choice had been made before lockdown and with no notion of the coming health crisis.

The much higher vulnerability to lockdown of the elderly is a striking finding of our study which may be related to this population’s greater concern about the risks associated with SARS-Cov-2 infection. This was not observed in other studies conducted among adults from UK [13] and older adults from Spain [20]. The increased risk for those aged 23–49 may be explained by higher exposure to the media and to social media, as was suggested by a large study conducted among 50,000 respondents from the general Chinese population [21]. Another study conducted during the SARS outbreak in Taiwan in 2007 also found a greater risk of developing depressive symptoms among nurses aged less than 35 [22]. The same results were found for the COVID-19 crisis among young adults in the US [23]. As regard to gender, we found that the proportion of women with impaired mental health during lockdown was much higher than for men, a result that was consistently observed in several other studies [21, 24–27].

As already reported in Italy [28], living conditions probably impacted undergraduate students’ mental health: the lack of outdoor space was associated with poorer self-rated mental health, and a smaller living space with depression symptoms. Although income level and type of housing (house or apartment) were not associated with impaired mental health in multivariate models, this is likely due to their correlation with other variables in the model such as outdoor space and housing size. Interestingly, those who were more used to spending time on screens and to spending less time outdoors seemed to have suffered less during lockdown. This observation, which we have not seen replicated in other studies, does not call into question the likely impact on anxiety, especially among young adults, of the increased exposure to screens during lockdown [29].

A relationship between self-rated physical and mental health was observed in our study as in another study conducted among adults from UK aged 50 years and more [30]. With respect to change, however, the lockdown period seemed to have had a symmetric impact on these two dimensions of health: while mental health globally deteriorated, a significant proportion of the respondents reported an improved physical health status. It is questionable whether lockdown allowed participants the time to take better care of their physical health, while their mental health status was impacted by being “locked up” at home.

With the two objectives of better understanding the events that most affected people in lockdown, and of confirming that the changes observed are related to the COVID-19 crisis and not to a secular trend, we conducted a separate analysis of the associations between mental health deterioration and a series of variables describing the events and perceptions related to the epidemic. All of them were associated with a poorer mental health status and increased depression and anxiety scores. Unsurprisingly, a COVID-19 diagnosis (for the participant or a relative) strongly impacted mental health. Anxiety as a consequence of the COVID-19 pandemic was also found in a study including 4793 parents and adolescents conducted in the UK during the first 6 weeks of lockdown [31]. Anxiety symptoms were also much more frequent among those with more screen time. A the same time, screen exposure has probably increased during lockdown as has been shown in both China and France [32, 33]. While it remains difficult to draw more detailed conclusions from such an intricate picture, those results suggest that at least part of the changes we observed were due to the crisis.

The decision to propose an online study in the MAVIE cohort was motivated by the need to quickly assess the actual consequences of lockdown and the risk factors for a potentially deteriorated mental health. It should, however, be kept in mind that, when compared to the French population, the participants had a higher level of education and that older adults were overrepresented [34]. Participants who enrolled were also probably more likely to be interested in HLIs, for many possible reasons, the main one being that they recently experienced an HLI (themselves or someone in the household). Further selection biases have to be considered as only a sub-sample of the cohort participants decided to answer our lockdown questionnaire. Table 1 clearly shows that they were different from those who did not respond: they were older, with a higher educational level, more income, living in a more populated area, less likely to be smokers, and more likely to drink alcohol, to use screens, to read, and to practice outdoor activities and sports. Although it is difficult to infer the impact that these selection biases may have had on our results, this may at least partly explain the surprising increased self-rated level of physical health measured during lockdown. It is also possible that people in poorer health were reluctant to answer the questionnaire because they were not comfortable with the situation. It is therefore likely that the impact of the crisis on the general population was greater than what we observed.

Also among the limitations, additional variables should have been added to our questionnaire, particularly in light of the results of other similar studies that, for example, showed an impact of lockdown on sleep disorders [7]. Similarly, an update of the level of psychotropic medicine use would have been useful too. Finally, the results of regression models should be interpreted with caution as no correction for multiple comparisons was used. This is in particular true for the weakes measures of association such the association between personal outdore space and self-perceived mental health.

Studies show that a change in the PHQ-9 score of 5 or more is considered clinically significant in the general population [35]. The fact that any change in score was chosen as a judging criterion in our study can therefore be discussed. Indeed, a small change may have no clinical impact on the participant, but the number of participants with a change greater than or equal to 5 was small (59 and 64 respondent reported such an increase in PHQ-9 and GAD-7 scores respectively). It would be however relevant to select this judgement criterion in analyses with a higher number of participants.

The observed decrease in the average self-reported levels of mental health and the increased anxiety symptoms may also have been partially due to a secular trend over the average 4.8 years that separated recruitment from the lockdown questionnaire. A notable change over such a short period is, however, unlikely and the strong correlation we found between self-reported concerns related to the epidemic and lower mental health indicators strongly argue in favour of a direct impact of the crisis. As an extra precaution, we assessed whether there was a link between the observed change and the duration time between the two questionnaires and found no significant trend.

## Conclusions

In conclusion, we prospectively identified an impact of lockdown on mental health among the participants of the French MAVIE cohort during the COVID-19 crisis. Women, younger and older people appeared to be the most vulnerable and facilitating living conditions such as the availability of an outdoor space and a sufficient inner living space clearly helped to maintain good mental health.

## Data Availability

Request for more information can be emailed to emmanuel.lagarde@u-bordeaux.fr.
